# Investigation of the Serum Level of Vitamin D in Patients with Ear Cholesteatoma

**DOI:** 10.22038/IJORL.2021.52513.2793

**Published:** 2022-03

**Authors:** Fatemeh Fanimolky, Maryam Amizadeh

**Affiliations:** 1 *Clinical Research Unit, Shafa Hospital, Kerman University of Medical Science, Kerman, Iran.*

**Keywords:** Cholesteatoma, Otitis media, Serum level of vitamin D

## Abstract

**Introduction:**

This study aimed to investigate the serum level of vitamin D in patients with ear cholesteatoma.

**Materials and Methods:**

This cross-sectional study was performed on 62 patients with middle ear cholesteatoma (case group) and 62 patients with simple chronic otitis media (control group). Both groups had the same age (32±1 in the case group and 34±1 in the control group; P=0.973) and gender. Vitamin D serum level was measured in the two groups. Data analysis was conducted using t-test and ANOVA.

**Results:**

According to the statistical analysis, a significant relationship was observed between the serum level of vitamin D and middle ear cholesteatoma (P=0.000). The results showed that the serum level of vitamin D was lower in the case group, compared to the control group.

**Conclusions:**

Vitamin D serum level was lower in the cholesteatoma group. Moreover, it was strongly associated with hearing loss, tinnitus, and vertigo.

## Introduction

Cholesteatomatous otitis media is considered a sort of cholesteatoma disease the morbidity of which is 0.5%1.8% (1). Most of the patients suffer from cholesteatomatous otitis media after adulthood. The patients aged 40 to 45 years old were the most ones suffering from this disease (2, 3). According to some researchers, auditory ossicles will be injured and the cholesteatoma extents unless otitis media is cured. With the development of cholesteatoma in the cranium, death of the patients will occur (4-6). There are no certain symptoms in the early stage of cholesteatoma. Similarly, its symptoms would be confused with the clinical symptoms of the simple otitis media (7).

Vitamin D (cholecalciferol) is defined as a hormone with a steroidal structure regulating calcium homeostasis and bone formation via reabsorption of kidneys, parathyroid glands, and bowel (8). Vitamin D serum level is considered different in the European, Middle East, and Asian countries (9). These differences are attributed to various factors, such as diet, air pollution, and limitation of exposure to sunlight (10). The normal blood level of vitamin D can be equal to or higher than 30 ng/ml; moreover, the blood level between 20 and 30 ng/ml is regarded as insufficient, and less than 20 ng/ml can be deficient (11). It is noteworthy that the high prevalence of vitamin D deficiency would be due to diet, as well as low exposure to sunlight (12).

 Little research has been carried out regarding the interrelation between vitamin D and various diseases. The role of vitamin D is to prevent respiratory infection, cochlear deafness, and demineralization of bone (13, 14). Some studies have also indicated that the low serum level of vitamin D increases chronic infections (15). Of these chronic diseases, the middle ear cholesteatoma can be mentioned. The middle ear cholesteatoma is categorized into four stages. In stage 1, only one of the middle ear spaces is involved with cholesteatoma. In stage 2, two or more spaces are involved; the third stage includes extra-cranial complications, and finally, the fourth stage includes intra-cranial complications. It is notable to say that the middle ear spaces contain attic, supratubal, sinotympan, as well as tympanic and mastoid cavities (16). Although many factors may cause cholesteatoma, serous otitis media can be considered an important factor (17). Considering different studies, vitamin D supplements are effective in improving the immune system and increasing anti-inflammatory effects (18). 

Seo Hwa Kim et al. (2014) reported that vitamin D inhibited the expression and activity of matrix metalloproteinase in human lung fibroblasts cells (19). 

In addition, Marianne Schmidt et al. middle ear cholesteatoma (20) likewise, Hitome Kobayashi et al. (2005) have indicated a reduction of vitamin D activity on matrix metalloproteinase production, which was made from Cholesteatoma Keratinocytes *in vitro* (21). However, few studies were carried out on vitamin D and cholesteatomatous intensity. Therefore, this study aimed at investigating the relationship between vitamin D serum level and the incidence of middle ear cholesteatoma, cholesteatoma extension, vertigo, hearing loss, and tinnitus. This made the present study novel research in its kind.

## Materials and Methods

This cross-sectional study attempted to assess vitamin D serum level in patients with ear cholesteatoma who were referred to the central hospital of Kerman, Iran, from January 2018 to September 2019. Kerman is located in the south-central part of Iran and based on the 2017 census, the population of Kerman was 821,394 in 221,389 households, making it the 10^th^ most populous city in Iran.

***Study Population***

The study population consisted of the patients who referred to the ear, nose, and throat (ENT) ward of a central hospital affiliated to Kerman University of Medical Sciences, Kerman, Iran, from January 2018 to September 2019. The inclusion criteria were the patients with the diagnosis of middle ear cholesteatoma and simple chronic otitis media. On the other hand, the cases who were taking vitamin D supplements, as well as those with a past medical history of middle ear surgery and sensory hearing loss were excluded from the study. The case group included patients (n=62) suffering from cholesteatoma, and 62 cases with simple chronic otitis media were assigned to the control group. It should be noted that there was no significant difference between the two groups in terms of gender and age.

**Study Design**

Sampling was performed in a full-census manner. After obtaining the informed consent, 5cc of blood was taken from each person, and the serum was separated by centrifugation and frozen at -20^°^C immediately. 

The frozen serum was sent to the laboratory as soon as possible in iceboxes. The IDS kit was used to measure vitamin D (US-made) that measured 25-hydroxyvitamin D in serum based on ELISA. 

Demographic characteristics (age, gender, family history of ENT disease, past medical history, and underlying diseases) were recorded in a checklist. Hearing assessment and tympanometry were conducted with Astra 2 device and the Zodiac model 901, respectively.

***Statistical Analysis***

To analyze the quantitative and qualitative variables, relative and absolute frequency were used. Following that, the obtained data were analyzed in SPSS software (version 20; IBM Inc., Chicago, IL, USA) through the chi-square and Fisher's exact tests for the frequencies. In addition, t-test and ANOVA were applied in both groups for the comparison of the continuous variables. 

All P-values were two-sided, and the significance level was set at P<0.05.

***Ethical Considerations***

The study protocol was approved by the Ethics Committee of Kerman University of Medical Sciences, Kerman, Iran (Code: IR.KUMS. REC.1396.111). The data of our manuscript is available.

## Results

**Fig 1:**
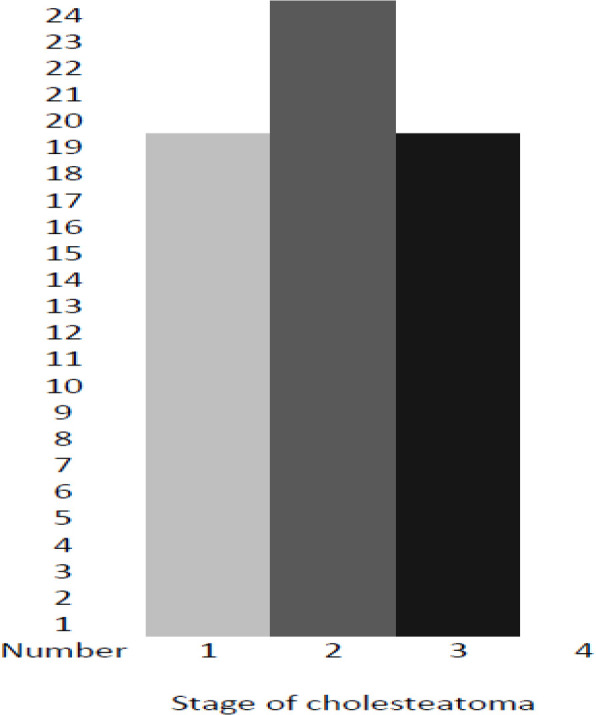
Stage of cholesteatoma

This study was carried out on 124 patients (the majority [n=74] of whom were male) suffering from middle ear cholesteatoma (n=62; case group) and simple chronic otitis media (n=62; control group). The clinical data of the patients were compared between the two groups in order to ensure that the results of the current research were accurate and credible. It should be noted that there was no significant difference between the groups in terms of gender, age, the presence or absence of immune system disorder, and place of living (P>0.05). Out of 124 participants, 37 males and 25 females participated in both case and control groups, respectively. The mean serum levels of vitamin D were 16.532 and 36.919 ng/ml in the case and control groups, respectively. The comparison revealed a significant relationship between vitamin D and cholesteatoma (P<0.0001) in statistical analysis. In other words, vitamin D was lower in the case group, compared to that in the control group. According to the classification of cholesteatoma (16), most of the participants were in stage 2, and no participant was observed in stage 4 (Figure1). Furthermore, the number of participants was equal in stages 1 and 3 (Table1).

**Table 1:** Demographic & baseline vitamin D and stage cholesteatoma in case and control analysis was done using t test

		**Case Group **	**Control Group**	**P value**	
Sex (male\female)		1.96	1.96	>0.999	
Age mean		32+/-1	34+/-1	.973	
vertigo +		56.45%	29.03%	0.94.	
Tinnitus +		69.35%	30.64%	.025	
Serum level of vitamin D					
Vitamin D mean		16.532ng/mL	36.91 ng/Ml	P value	
Vitamin D normal		n=19	n=42	<0.001	
Vitamin D insufficient		n=18	n=9	<0.001	
Vitamin D deficient		n=25	n=8	<0.001	
Stage cholesteatoma					
		Stage of cholesteatoma	Stage I: n=19	
		Stage II: n=24	
		Stage III: n=19	
		Stage IV: n=0	


The mean serum level of vitamin D was evaluated in different stages. In stages 1, 2, and 3, the mean values of vitamin D3 were 16.25, 16.81, and 16.51 ng/ml, respectively. In the statistical analysis, there was no significant relationship between the serum level of vitamin D and the stage (P=0.808). However, a significant relationship was observed between the hearing loss severity and serum level of vitamin D (P<0.0001). The results showed that 59 subjects were diagnosed with mild hearing loss, 56 cases had moderate hearing loss, and 9 patients suffered from severe hearing loss. The mean serum level of vitamin D was assessed in each of these auditory levels. 

In the mild, moderate, and severe levels, the mean serum levels of vitamin D were 33.20, 22.25, and 17.3 ng/ml, respectively. According to the American Speech-Language-Hearing Association (22), five hearing levels include 1) mild hearing loss (25-40 dB), 2) moderate hearing loss (40-55 dB), 3) moderately severe hearing loss (55-70 dB), 4) severe hearing loss (70-90 dB), and 5) profound hearing loss (more than 90 dB). 

It was interpreted that those with more severe hearing loss had a lower mean serum level of vitamin D. It is noteworthy that the investigation of the serum level of vitamin D and its correlation with the severity of hearing loss in the two groups demonstrated that the serum level of vitamin D obtained less in every three subtitles of hearing loss in the case group, compared to the control group. (Table 2).

**Table2:** Serum level of Vitamin D and severity of hearing loss analysis was done using Anova test

**Severity of hearing loss**	**Vit. D mean in case**	**Vit. D mean in control**	**P value**
Mild hearing loss (n=59)	18.75+\-7.84ng/mL	36.72+\-11.8 ng/mL	**0.001**
Moderate hearing loss (n=56)	15.98+\-10.96 ng/mL	37.50 +\- 6.92ng/mL	**0.001**
Moderately Severe hearing loss(n=9)	16.10+\-7.81ng/mL	………	**……**

 Meanwhile, the investigation of the serum level of vitamin D and its association with tinnitus in both groups demonstrated that the mean serum level of vitamin D was lower in the case group, compared to the control group. In addition, there was a significant relationship between the two groups (Table 3). 

**Table 3:** serum level of vitamin D and tinnitus analysis was done using Anova test

	**Vitamin D3 mean in case**	**Vitamin D3 mean in control**	**P value**
Tinnitus Group(n=62)	15.53+\-7.55 ng.ml	36.10+\-9. 29ng.ml	0.001
Non-tinnitus Group(n=62)	Type 16.98+\-10.80 ng.ml	38.65+\-13.40 ng.ml	0.001

Meanwhile, 53 cases out of 124 participants of the study complained of vertigo. The mean serum levels of vitamin D in vertigo and non-vertigo groups were 27.78 and 27.29 ng/ml, respectively. No significant relationship was observed between the serum level of vitamin D and vertigo (P=0.94).

## Discussion

The current study aimed to investigate the clinical data on 62 patients with cholesteatoma and 62 cases with simple otitis media. 

The serum levels of vitamin D in the patients in both groups were analyzed and compared in this study. The correlation of serum level of vitamin D with the incidence of cholesteatoma, vertigo, tinnitus, stage of cholesteatoma, and severity of hearing loss was also investigated. It was found that the serum level of vitamin D in the patients with cholesteatoma was lower than that in the control group with simple otitis media. According to the reports by Kobayashi H et al. (2005), increasing the serum level of vitamin D protects the middle ear from cholesteatoma via methyl proteinase reduction (21). Furthermore, Marianne Schmidt et al. (2001) have indicated that the up-regulation of matrix metalloprotease-9 plays a key role in the pathogenesis of middle ear cholesteatoma (20). It is noteworthy that these results are in line with the findings of the present study. The serum level of vitamin D in the participants with more severe hearing loss was significantly lower than that in the participants with less hearing loss. On the other hand, the serum level of vitamin D in cases with tinnitus was dramatically low, compared to participants without tinnitus. Based on a study conducted by Piers Dawes et al. (2020), the findings revealed that increasing vitamin D in the diet resulted in the reduction of hearing problems (23). 

Similarly, Isil Karaer et al. (2019) indicated that a reduction in the serum levels of vitamin D caused cochlear damage (24). It is noteworthy that these results are in line with the findings of the present study. In a meta-analysis, Mohammed A. Al Garni et al. (2018) pointed out that the reduction of the serum level of vitamin D resulted in benign paroxysmal positional vertigo (25). 

The results were in line with those obtained by Jing Ding et al. (2019) (26). However, different findings were reached in the current study, and the reason would be the small sample size of the case group, compared to that in the aforementioned studies. 

***Limitations of the Study***

Regarding the limitation of this study, one can refer to the low cooperation of the cases. Another limitation was the lack of access to the background of the cases' vitamin D levels. Moreover, due to cholesteatoma progress in years, it could be better to design a prospective study rather than a cross-sectional study. 

## Conclusion

The serum level of vitamin D was lower in the cholesteatoma group, and it was strongly correlated with hearing loss, tinnitus, and vertigo. It seems that the vitamin D serum level could prevent the formation and extension of cholesteatoma, which can be considered crucial for the patients.
